# Semaglutide-induced fixed drug eruption

**DOI:** 10.1016/j.jdcr.2024.10.012

**Published:** 2024-11-02

**Authors:** Sahithi Talasila, Shayan Waseh, Jeffrey Y. Liu, Ibrahim Khalifeh, Afton R. Metkowski, Sylvia Hsu

**Affiliations:** a Sidney Kimmel Medical College, Thomas Jefferson University, Philadelphia, Pennsylvania; b Department of Dermatology, Temple University Lewis Katz School of Medicine, Philadelphia, Pennsylvania; c Department of Pathology, Temple University Lewis Katz School of Medicine, Philadelphia, Pennsylvania

**Keywords:** adverse drug reaction, drug hypersensitivity, FDE, fixed drug eruption, GLP-1 receptor agonist, Ozempic, semaglutide, type 2 diabetes

## Introduction

Fixed drug eruption (FDE) represents a relatively common medication-induced hypersensitivity reaction, characterized by recurrent hyperpigmented to erythematous, strikingly round patches, which may occasionally exhibit bullae formation or become generalized.^1^^,^^2^

We present a case of a 62-year-old woman with clinicopathologic features consistent with FDE secondary to injections of semaglutide (Ozempic, Novo Nordisk, Bagsværd, Denmark), a glucagon-like peptide 1 (GLP-1) receptor agonist used as an adjunct to diet and exercise to improve glycemic control and to reduce the risk of major adverse cardiovascular events in adults with type 2 diabetes mellitus (DM). To our knowledge, this is the first reported case of FDE associated with semaglutide to date.

## Case report

A 62-year-old woman with a history of type l DM, obesity, and hypertension presented to the clinic for evaluation of persistent round hyperpigmented patches on the buttock and abdomen that had progressively appeared during the past 3 months. The patient reported that the lesions first appeared within 72 hours of initiating semaglutide therapy, consisting of weekly 2 mg subcutaneous injections for management of her DM. The patient reported the onset of erythema and pruritus on the buttock and abdomen within 24-48 hours following semaglutide injections, despite the injections being administered at distant anatomical sites. These initially erythematous and pruritic reactions subsequently progressed into asymptomatic chronic hyperpigmented patches with an erythematous border. The patient denied any recent changes to her medications, which was corroborated by her pharmacy dispense record. She also denied any new over-the-counter supplements and medications, including nonsteroidal anti-inflammatory drugs.

**Fig 1:**
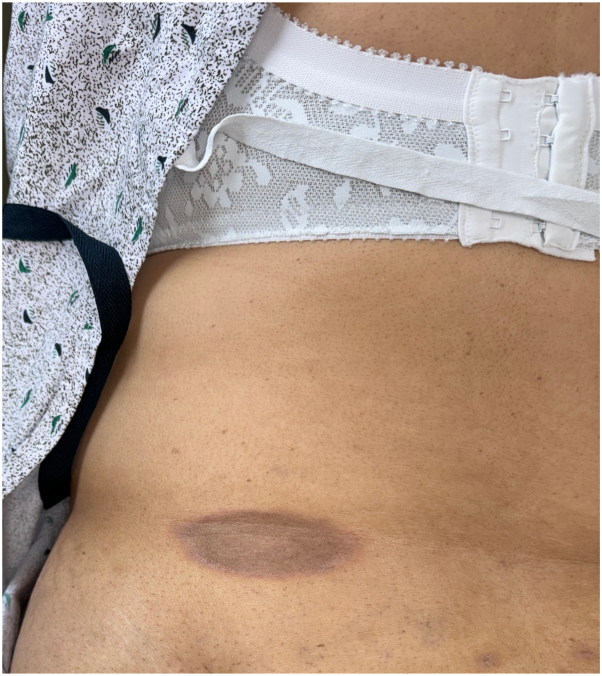
FDE on left lower back presenting as a dusky, violaceous, hyperpigmented, well-circumscribed *oval* patch with erythematous border. Similar lesions were present on the buttock and abdomen. *FDE*, Fixed drug eruption.

**Fig 2:**
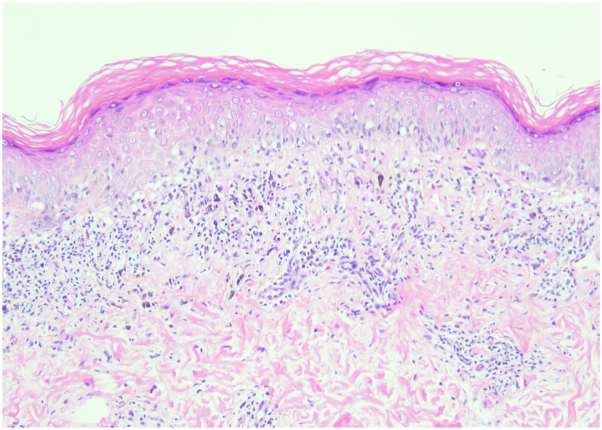
Lichenoid interface dermatitis with significant pigment incontinence. Numerous necrotic keratinocytes were noted throughout the epidermis with conspicuous pigment incontinence seen throughout the superficial dermis (hematoxylin and eosin 100×).

On physical examination, the patient had multiple dusky hyperpigmented round patches with erythematous borders on her buttock and abdomen (Fig 1). The lesions were sharply demarcated, consistent with FDE. No evidence of mucocutaneous involvement was observed. A 4-mm punch biopsy was performed on a lesion on the right buttock, and histopathological examination revealed lichenoid interface dermatitis, consistent with an FDE (Fig 2). Numerous necrotic keratinocytes were noted throughout the epidermis with conspicuous pigment incontinence seen throughout the superficial dermis.

Semaglutide was subsequently discontinued in collaboration with the patient's primary care physician. On follow-up at 1 month, no new lesions were noted, and the patient's initial lesions were completely hyperpigmented with no erythematous border, consistent with postinflammatory hyperpigmentation secondary to resolving FDE. The patient was instructed that this postinflammatory hyperpigmentation may take many months to fully resolve.

## Discussion

Semaglutide is a GLP-1 receptor agonist approved for the treatment of type 2 DM. It enhances glucose-dependent insulin secretion, reduces inappropriate glucagon secretion, and slows gastric emptying, thereby improving glycemic control.^3^ Beyond its approved indications, semaglutide is also used off-label for weight management in individuals with obesity and for the treatment of prediabetes. While generally well tolerated, semaglutide has been associated with adverse reactions, including gastrointestinal disturbances and hypersensitivity reactions.^4^

FDE was not reported during clinical trials for semaglutide, although drug-induced hypersensitivity reactions, such as generalized pruritus and rash, have been documented.^4^ These hypersensitivity reactions differ significantly from the FDE described in this report. FDE is a delayed hypersensitivity reaction characterized by well-defined, recurrent patches at discrete sites, driven by localized immune responses involving drug-specific cytotoxic T cells retained in the skin.

FDE typically manifests within days of initial drug exposure and recur within 24 hours upon re-exposure, often presenting as well-demarcated, violaceous patches or plaques that leave postinflammatory hyperpigmentation after resolution. The most frequently implicated drugs in FDE include antibacterial agents (eg, trimethoprim-sulfamethoxazole, tetracyclines), nonsteroidal anti-inflammatory drugs (eg, ibuprofen, naproxen), acetaminophen, and anticonvulsants.^5^ The pathogenesis of FDE is not fully understood, but it is hypothesized to involve the activation of intraepidermal CD8+ T cells, which persist in the skin even after the drug is discontinued. Upon re-exposure to the drug, these cells rapidly release cytotoxic mediators, causing localized tissue damage.^5^ FDE is more commonly associated with oral medications. Reports of FDE induced by parenteral agents, including biologicals and other injectable medications, are less common but have been documented.^6^

To our knowledge, this is the first reported case of semaglutide-induced FDE. The clinical presentation observed in this patient highlights the importance of considering FDE in patients presenting with recurrent, well-demarcated lesions following the administration of semaglutide or other GLP-1 receptor agonists. Clinicians should maintain a high index of suspicion and advise patients to report any unusual dermatologic reactions during treatment. If FDE is suspected, discontinuation of the offending agent and exploration of alternative therapies should be considered.

## Conflicts of interest

None disclosed.
